# Association between perinatal intimate partner violence and breastfeeding outcomes: a systematic review and meta-analysis

**DOI:** 10.1186/s12884-026-09737-3

**Published:** 2026-07-27

**Authors:** Zelalem Nigussie Azene, Berihun Dachew, Mehari Woldemariam Merid, Nicole Reilly, Catherine MacPhail

**Affiliations:** 1 School of Medical, Indigenous and Health Sciences, Faculty of Science, Medicine and Health, University of Wollongong, Northfields Avenue, Wollongong, NSW 2522 Australia; 2 Department of Women’s and Family Health, School of Midwifery, College of Medicine and Health Sciences, University of Gondar, Gondar, Ethiopia; 3 School of Population Health, Curtin University, Perth, WA Australia; 4 The Florey Institute of Neuroscience and Mental Health, University of Melbourne, 30 Royal Parade, Parkville, VIC 3052 Australia; 5 Department of Epidemiology and Biostatistics, Institute of Public Health, College of Medicine and Health Sciences, University of Gondar, P.O. Box 196, Gondar, Ethiopia; 6 Graduate School of Medicine, Faculty of Science, Medicine and Health, University of Wollongong, Wollongong, Australia; 7 Discipline of Psychiatry and Mental Health, School of Clinical Medicine, UNSW Sydney and St John of God Burwood Hospital, Sydney, Australia

**Keywords:** Breastfeeding outcomes, Intimate partner violence, Systematic review and meta-analysis

## Abstract

**Background:**

Intimate partner violence (IPV) negatively affects the health of both the mother and the child. These risks are particularly critical during the perinatal period, a time of heightened vulnerability. However, the relationship between maternal IPV exposure and breastfeeding outcomes remains uncertain, and existing findings are inconsistent and inconclusive. Therefore, this systematic review and meta-analysis aimed to synthesise the existing evidence on the association between perinatal IPV and breastfeeding outcomes.

**Methods:**

A systematic literature search was conducted using relevant databases between June 15 and August 2, 2024. All peer-reviewed observational studies that specifically investigated the link between Perinatal (P)-IPV and breastfeeding outcomes, such as early initiation, exclusivity, and duration among the perinatal population, were included. Heterogeneity across the included studies was assessed using the Cochrane Q test and I² statistics. Publication bias was assessed through funnel plots and Egger’s test. Finally, a random effects model was used to estimate the pooled adjusted odds ratios and their 95% CIs.

**Results:**

Nineteen observational studies published between 2007 and 2024, comprising 347,706 mother-child pairs, were included in the review. Of these, 17 studies were eligible for the meta-analysis. A meta-analysis of nine studies indicated that P-IPV was associated with a 36% higher odds of delayed initiation of breastfeeding (AOR: 1.36; 95% CI: 1.14–1.63), with both physical (AOR: 1.20; 95% CI: 1.02–1.42) and psychological P-IPV (AOR: 1.38; 95% CI: 1.09–1.74) were associated with higher odds of not initiating breastfeeding within one hour of birth. Similarly, women exposed to P-IPV had higher odds of not exclusively breastfeeding (EBF) at six months (AOR: 1.50; 95% CI: 1.21–1.85), with similar associations observed for both physical (AOR: 1.52; 95% CI: 1.18–1.95) and psychological P-IPV (AOR: 1.56; 95% CI: 1.39–1.76).

**Conclusions:**

This review found that P-IPV was associated with delayed breastfeeding initiation and a lower likelihood of EBF at six months. The findings highlight the potential value of trauma-informed and trauma-sensitive screening, support, and referral pathways for women experiencing P-IPV.

**Supplementary Information:**

The online version contains supplementary material available at https://doi.org/10.1186/s12884-026-09737-3.

## Introduction

Intimate partner violence (IPV) is a widespread public health issue, disproportionately affecting women worldwide [1–3]. The World Health Organisation (WHO) defines IPV as any behaviour within an intimate relationship that causes physical, psychological, or sexual harm. This includes: acts of physical aggression, sexual coercion, psychological abuse, and controlling behaviours [4]. IPV has far-reaching consequences, extending beyond immediate physical and psychological harm, posing substantial risks to both maternal and child health outcomes [5–7]. These risks are particularly critical during the perinatal period, when women may be biologically and psychosocially vulnerable [8, 9]. Global estimates suggest that perinatal (P)- IPV affects 3.7% to 66.9% of women, with psychological violence being the most prevalent type [10]. A recent study involving 601,534 women from 57 low- and middle-income countries found that 6.3% experienced IPV during pregnancy, with significant disparities across countries and regions [11].

P-IPV has been linked to several maternal health consequences, including physical injuries, sexually transmitted infections (STIs), and pregnancy and childbirth complications [1, 12]. Additionally, P-IPV is strongly associated with adverse mental health outcomes, such as depression, anxiety, and post-traumatic stress disorder (PTSD) among survivors [6, 13–15]. A study in the United States (USA) found that IPV-exposed women exhibited elevated levels of TNF-α, a pro-inflammatory cytokine, during pregnancy [16], suggesting a biological mechanism contributing to adverse health outcomes in women. IPV during pregnancy is linked to harmful health behaviours, including smoking and alcohol consumption [16, 17]. P-IPV can also hinder a woman’s ability to access and utilise healthcare services [18], potentially delaying or preventing essential interventions for pregnancy-related complications. Evidence also indicates that women experiencing P-IPV often face obstacles in seeking support and may find it difficult to access proper health and social care services, which can worsen the effects of violence during the perinatal period [19]. These adverse consequences may contribute to poorer maternal health outcomes; for example, an Australian study revealed that women exposed to P-IPV were more likely to experience hospital admissions for threatened preterm labor [20].

The effects of P-IPV extend beyond maternal health and can profoundly impact infant and child health outcomes. It is consistently linked to adverse birth outcomes, such as preterm birth, low birth weight (LBW), and small for gestational age (SGA) infants [12, 15, 17, 21]. P-IPV can also disrupt mother-infant bonding, interfere with breastfeeding and parenting practices, increasing the risk of long-term developmental and behavioural challenges in children [2, 22].

Breast milk provides essential nutrients and bioactive compounds that promote infant growth, immune function, and neurodevelopment [23–25]. It also contains protective agents like immunoglobulins and cytokines [24]. Exclusive breastfeeding (EBF) reduces childhood illnesses and infections [24, 26, 27] and benefits mothers by lowering the risk of postpartum complications, chronic diseases, and certain cancers [24, 28, 29].

The WHO recommends initiating breastfeeding within the first hour after birth, exclusively breastfeeding for the first six months, and continuing breastfeeding for at least two years [30]. However, global adherence to breastfeeding recommendations remains suboptimal [31, 32]. Globally, only 42% of newborns are breastfed within the first hour of birth, and only 41% are exclusively breastfed for the first six months, which is below the 2030 target of 70%. Furthermore, 71% of children continue to be breastfed for at least one year of age, but this rate falls to 45% by the child’s second year [33]. A study from Ethiopia found that among women exposed to antenatal intimate partner violence, 59.3% initiated breastfeeding within the first hour after birth, and 70.5% exclusively breastfed their infants during the first six weeks after birth [34]. IPV can further impact breastfeeding practices by affecting maternal physical and mental health, reducing access to support, and disrupting the mother-infant bond [35, 36].

Evidence on the association between P-IPV and breastfeeding outcomes is inconsistent across studies. Some studies report that women exposed to IPV are less likely to initiate breastfeeding [37–42], whereas others show null associations [39, 43–46]. Likewise, findings on EBF are mixed. While some studies suggest that P-IPV is associated with early cessation and reduced duration of EBF [37, 45, 47, 48], other studies report no significant association between P-IPV and EBF or overall breastfeeding duration [43, 47, 49–51].

This systematic review and meta-analysis is therefore aimed at comprehensively synthesising the existing evidence on the link between women’s exposure to IPV during the perinatal period and breastfeeding outcomes. Previously, three systematic reviews assessed the link between IPV and breastfeeding, focusing on studies published between 2000 and 2023 [52–54]. However, none of those reviews have conducted a meta-analysis or performed any quantitative synthesis. Additionally, one of the reviews incorrectly applied the Strengthening the Reporting of Observational Studies in Epidemiology (STROBE) guidelines as a quality assessment tool [53]. STROBE is intended to enhance the reporting of observational studies, not to evaluate their quality [55]. Furthermore, recent literature has emerged on this topic.

## Methods

### Protocol registration and reporting

This systematic review and meta-analysis was reported according to the guidelines set forth by the Preferred Reporting Items for Systematic Reviews and Meta-Analyses (PRISMA) of observational studies [56] (Table S1). The study protocol was registered with the International Prospective Register of Systematic Reviews (PROSPERO), registration number (CRD42024555048), and published in PLOS ONE [57].

### Search strategy and databases

The search strategy was developed using the PICO framework (Population: women in the perinatal period, Intervention: exposure to IPV, Comparison: women not exposed to IPV, and Outcomes: Breastfeeding outcomes - initiation, duration, and exclusivity) [58]. We conducted an initial scoping search to identify relevant keywords and subject headings specific to each database and pilot-tested the search strategy in Medline, Scopus, and CINAHL to refine keywords and Boolean operators. The search strategy was peer-reviewed by professional medical/academic librarians who are experts in health-based systematic reviews to ensure comprehensiveness and accuracy in capturing relevant literature across the selected databases and sources. The search was limited to studies conducted on humans and published in English. We updated the search during the review manuscript preparation process to include new studies as they became available.

Due to their comprehensive coverage of health, medicine, nursing, and psychological literature, nine electronic databases were searched from their inception until August 2, 2024, without geographic restrictions. These included: PsycInfo, Scopus, Web of Science, Medline, Cochrane, JBI EBP, CINAHL, Informit, and PubMed.

Each database was searched for the concepts of ‘Intimate Partner Violence’ and ‘Breastfeeding’ using a wide range of potential search terms, including Medical Subject Headings (MeSH) terms and keywords. We manually searched the reference lists of the included articles to identify and include any additional relevant studies. We updated the search on March 23, 2025, and did not identify any additional primary studies meeting our inclusion criteria, but we did find one relevant systematic review published on September 1, 2024 [54]. The search strategies for the respective databases are in the supplementary data (**S2 File**).

### Eligibility criteria

All peer-reviewed primary observational studies that specifically investigated the association between P-IPV and breastfeeding outcomes, such as early initiation, exclusivity, and duration, in the perinatal population were included. No restrictions were placed on publication date during the search and study selection process. We excluded non-peer-reviewed studies, such as case reports, case series, editorials, letters to the editor, commentaries, and opinion pieces without original research data, as well as secondary or review articles. In addition, we excluded studies focusing on populations other than women in the perinatal period.

### Exposure variable: P-IPV

An IPV that occurred during the perinatal period was our exposure of interest, defined as any behaviour within a current or former intimate relationship with a male that causes physical, sexual, or psychological harm, including acts of physical aggression, sexual coercion, psychological abuse, economic abuse (restricting access to financial resources, bank accounts or credit cards, education, employment and health care), controlling behaviour (restricting the victim’s contact with family, friends, and social networks, constantly checking on the victim’s whereabouts, communications, and activities, manipulation, regulating daily life) [59, 60]. Physical violence includes intentional acts of physical force or aggression such as pushing, slapping, throwing, hair pulling, punching, kicking, or burning, and the use of a weapon, perpetrated with the potential to cause harm, injury, disability, or death. Emotional/psychological violence is defined as acts or threats of acts, such as shouting, controlling, intimidating, humiliating, and threatening the victim. Sexual violence is defined as the use of force, coercion, or psychological intimidation to force a woman to engage in a sex act against her will, whether or not it is completed. Since IPV measurements or screening tools typically evaluate violence over the past year and lifetime [61], studies on P-IPV vary in the timeframes they cover. To gather more comprehensive data demonstrating the risks of P-IPV, we define P-IPV in this paper as an abuse experience occurring 12 months before conception, throughout pregnancy, and up to 12 months after childbirth [1, 62].

### Outcomes: Breastfeeding outcomes

The primary outcomes of this review were the first three core Infant and Young Child Feeding (IYCF) indicators established by the WHO: (1) the early initiation of breastfeeding, (2) EBF under 6 months, and (3) continued breastfeeding at least 2 years or beyond [63].

Early initiation of breastfeeding was defined as the proportion of children who were put to the breast within one hour of birth. EBF was measured as the proportion of infants aged 0–6 months who were fed exclusively with breast milk during the previous day and given no other foods or beverages except medication. Continued breastfeeding was measured as the proportion of women who breastfed for up to 2 years or beyond.

### Setting

Studies conducted in any geographical location, with no restrictions on region or country, and people of all ethnicities and races were included.

### Study identification and screening

The retrieved studies from the search were uploaded to Covidence (Veritas Health Information, Melbourne, Australia), an online software. Duplicates were identified and removed, and the titles and abstracts, and full-text reviews were independently screened by two reviewers (ZNA and CM) [64]. Studies on which both reviewers agreed were either excluded or progressed to the next stage. Disagreements were resolved through consensus or by involving a third reviewer (LGS). The study inclusion process is illustrated in a flow diagram adapted from the PRISMA guidelines [65].

### Data extraction and management

Data were extracted from all included studies using Covidence [64] and were independently performed by two reviewers. All reviewers independently extracted three articles to pilot-test the data extraction tool and process. Following this, ZNA extracted all studies, while CM and LGS each independently extracted half of the studies. Any discrepancies between reviewers were resolved through discussion, and when consensus could not be reached, the reviewer not involved in the study extraction (CM or LGS) served as the third reviewer.

The following information was extracted; title, prevalence of IPV, sample size, type of IPV exposure, timing of IPV exposure, tool to measure IPV, outcome measures, definition of breastfeeding outcomes, lead author name and email address, publication year, country in which the study was conducted (developmental status), comparison group, setting, study characteristics (study design, effect measures, effect estimate type and value, confidence Intervals (CI), p-values, standard error, confounders included in the analysis, start date and end date of the study, study funding sources, possible conflicts of interest for study authors), population characteristics (mean age, age range and standard deviation (SD), ethnicity, socioeconomic status, education level, relationship status, type of antenatal care, gravidity, parity, method of recruitment) (Excel File S3).

### Risk of bias (Quality) assessment

We assessed the methodological quality (risk of bias) of the included studies using a standardised, study-design-specific critical appraisal tool developed by the Joanna Briggs Institute (JBI). The tool comprises eight items for analytical cross-sectional studies and 11 for cohort studies with response options of ‘Yes,’ ‘No,’ ‘Unclear,’ and ‘Not Applicable (NA).’ Two reviewers (ZNA and CM) independently assessed the quality of the included studies and compared their results. Any discrepancies were resolved through discussion and consensus; if agreement could not be reached, a third reviewer (MWM) was consulted for the final decision. Studies were classified based on the percentage of ‘Yes’ responses to the questions aligned with the critical appraisal tool specific to the study design. Hence, those studies with ≥ 70% of “Yes” responses were labelled as high quality (low risk of bias), 50–69% were categorised as moderate quality (moderate risk of bias), and those with < 50% were deemed low quality (high risk of bias) [66, 67]. To inform our quality assessment of exchangeability, we developed a priori Directed Acyclic Graph (DAG) using DAGitty software. The DAG was constructed using evidence from the relevant literature and subject-matter knowledge on determinants of P-IPV and breastfeeding outcomes. Relevant variables were identified and incorporated into the DAG, and causal relationships were specified based on established evidence and theoretical assumptions. The resulting DAG was used to identify a minimally sufficient adjustment set for confounding control. We then assessed whether the included studies adjusted for these key confounders, which informed our judgment of the risk of confounding bias. The DAG figure has been published elsewhere [34].

### Data analysis

A meta-analysis was performed using Stata™ Version 17. A random-effects meta-analysis model was employed to estimate the pooled effect size. Effect sizes were predominantly reported as adjusted odds ratios (AORs) with corresponding 95% confidence intervals (CIs) across the included studies. However, two studies presented their effect estimates as adjusted hazard ratios (AHR), and one study reported a crude odds ratio (COR). The study reporting the COR was retained in the meta-analysis, while effect estimates from the two studies reporting AHR were pooled separately.

In this meta-analysis, women not exposed to IPV during the perinatal period were considered as a reference (control) category. However, some studies reported AOR by considering women exposed to IPV during the perinatal period as a reference category. To maintain consistency and ensure uniform directionality of effect estimates across studies, the reported AORs and their corresponding 95% CIs were recalculated by taking their reciprocals.

### Heterogeneity and publication bias assessment

The presence of heterogeneity across the included studies was assessed using the I² statistic, with thresholds of 25%, 50%, and 75% interpreted as low, moderate, and high heterogeneity, respectively; an I² of 0% indicates no observed heterogeneity [68]. Potential publication bias was evaluated using visual inspection of the funnel plot for asymmetry and Egger’s test at a 5% significance level.

## Results

### Study identification and selection

**Fig. 1:**
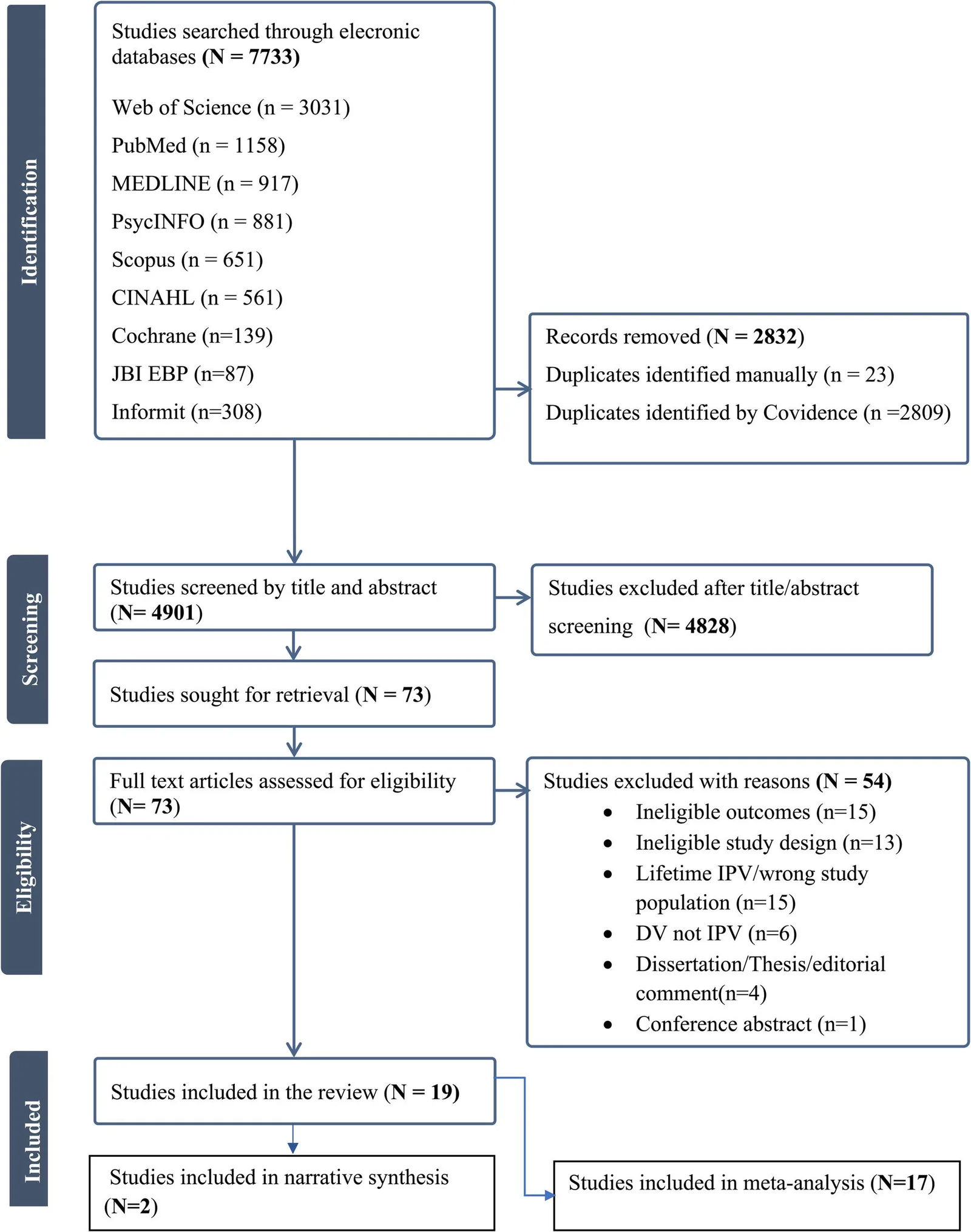
Flow chart of study selection for the systematic review and meta-analysis of P-IPV and breastfeeding outcomes

A total of 7,733 records were retrieved from databases and registers. Figure 1 shows the flow of studies through the review process. After removing 2,832 duplicates (2,809 identified by Covidence and 23 manually), 4,901 records remained for the title and abstract screening. Following title and abstract screening, 4,828 records were excluded, leaving 73 articles for full-text review. Of these, 54 were excluded based on eligibility criteria, resulting in 19 studies included in the systematic review, of which 17 studies were eligible for the meta-analysis.

### Characteristics of the included studies

Table 1 presents the characteristics of the included studies. A total of 19 studies, comprising 347,706 study participants, were included in this review. Of which 11 were cross-sectional [6, 40, 44, 47, 50, 69–74] and eight were longitudinal cohort studies [21, 42, 43, 45, 75–78]. These studies were published between 2007 and 2024. Sample sizes in individual studies varied widely, ranging from 101 to 195,264, with a median of 1,191 participants.

Geographically, the majority of studies (*n* = 12) were conducted in low- and middle-income countries [6, 44, 50, 69–72, 74–78], while seven were from high-income countries [21, 40, 42, 43, 45, 47, 73]. Among low- and middle-income countries, three studies were conducted in Africa [44, 74, 78] (two in Nigeria and one in Tanzania), five in South America [50, 69, 70, 75, 77] (four in Brazil and one in Colombia), and four in Asia [6, 71, 72, 76] (two in Bangladesh, one in Vietnam, and one in Pakistan). Among the high-income countries, two studies were from Australia [21, 43], three from the USA [45, 47, 73], one from Hong Kong [40], and one from Spain [42].

### P-IPV exposures

Most studies examined the three common types of P-IPV: physical, sexual, and psychological/emotional violence [40, 42–45, 70–72, 74, 77, 78]. Six studies focused solely on physical P-IPV [21, 47, 50, 69, 73, 75], while two studies assessed P-IPV as ‘any P-IPV’ without distinguishing specific types [6, 76] (Table 1).

**Table 1:** Key characteristics of included studies on P-IPV and breastfeeding outcomes

Author (s), (publication year)	Country	Sample size	Study design	IPV type	Periods of IPV	BF outcomes	Effect estimate	Statistical significance, *P*-Value	Key findings
Aristizabal et al. (2022) [69]	Colombia	11,416	Cross-sectional	Physical	During pregnancy	BFI and EBF	BFI = 1.08 EBF = 1.16	No, NR	No statistically significant relationship was observed with any of the breastfeeding indicators.
Ariyo et al. (2021) [74]	Nigeria	2465	Cross-sectional	Physical, sexual, and Psychological/ Emotional	During pregnancy; postpartum	EBF	1.35	Yes, NR	P-IPV is significantly associated with reduced likelihood of EBF practice.
Chaves et al. (2019) [21]	Australia	17,564	Cohort	Physical	Pre-pregnancy; during pregnancy	BFI and EBF	BFI = 1.65 EBF = 1.71	Yes, < 0.001	P-IPV was significantly associated with delayed initiation of breastfeeding and reduced EBF.
Do et al. (2022) [76]	Vietnam	148	Cohort	Any	During pregnancy	EBF	3.33	Yes, NR	Exposure to P-IPV was significantly associated with reduced practice of EBF.
Hasselmann et al. (2016) [75]	Brazil	564	Cohort	Physical	Pre-pregnancy; during pregnancy; postpartum	EBF	1.23	No, NR	No significant relationship was found between P-IPV exposure and the practice of EBF.
Islam et al. (2017) [71]	Bangladesh	426	Cross-sectional	Physical, sexual, and Psychological /Emotional	Postpartum	EBF	5.88	Yes, NR	Women who experienced physical IPV and psychological IPV after childbirth were significantly less likely to exclusively breastfeed their infants.
James et al. (2014) [43]	Australia	2111	Cohort	Physical, sexual, and Psychological /Emotional	Postpartum	BFD	1.25	No, -0.25	Findings suggest that women who experience P-IPV are just as likely to breastfeed as the broader population of women.
Khalid et al. (2024) [72]	Pakistan	1191	Cross-sectional	Physical, sexual, and Psychological /Emotional	During pregnancy; Postpartum	BFI and EBF	BFI = 1.37 EBF = 1.28	Yes, NR	Logistic regression showed that women who experienced physical or emotional P-IPV were less likely to exclusively breastfeed.
KjerulffMadsen et al. (2019) [78]	Tanzania	1128	Cohort	Physical, sexual, and Psychological /Emotional	During pregnancy	EBF	2.87	Yes, NR	Women exposed to P-IPV had higher odds of early termination of EBF, and those exposed to all three P-IPV types had twice the odds compared to unexposed women.
Lau et al. (2007) [40]	Hong Kong	1200	Cross-sectional	Physical, sexual, and Psychological /Emotional	During pregnancy	BFI	1.85	Yes, NR	P-IPV was significantly associated with delayed initiation of breastfeeding.
MartindeLasHeras et al. (2019) [42]	Spain	779	Cohort	Physical, sexual, and Psychological /Emotional	Pre-pregnancy; during pregnancy	BFI	2.00	Yes, NR	Mothers exposed to psychological P-IPV had higher odds of delayed breastfeeding initiation.
Miller-Graff et al. (2018) [45]	USA	101	Cohort	Physical, sexual, and Psychological /Emotional	During pregnancy; postpartum	BFI, EBF and BFD	BFI = 1.61 EBF = 2.44 BFD = 4.55	Yes, for BFD, but No for BFI and EBF P-value NR	P-IPV was not significantly associated with delayed breastfeeding initiation or early termination of EBF, but it was significantly associated with reduced breastfeeding duration.
Moraes et al. (2011) [50]	Brazil	811	Cross-sectional	Physical	During pregnancy	EBF	1.30	Yes, NR	A significant association was found between physical P-IPV and early discontinuation of EBF.
Obioha et al. (2022) [73]	USA	105,230	Cross-sectional	Physical	Pre-pregnancy; during pregnancy	BFI and BFD	BFI = 1.10 BFD = 1.18	No, NR	Exposure to physical P-IPV was not significantly associated with early termination of EBF or delayed initiation of breastfeeding.
Olubodun et al. (2023) [44]	Nigeria	3749	Cross-sectional	Physical, sexual, and Psychological /Emotional	Pre-pregnancy; during pregnancy; postpartum	EBF	1.09	No, NR	No significant association was found between any form of P-IPV and EBF.
RibeiroMRC et al. (2021) [77]	Brazil	1146	Cohort	Physical, sexual, and Psychological /Emotional	Pre-pregnancy; during pregnancy	EBF	1.05	No, NR	The adjusted Cox regression showed no significant association in EBF duration between mothers exposed to P-IPV and those not exposed.
Tran et al. (2020) [6]	Bangladesh	2000	Cross-sectional	Physical, sexual, and Psychological /Emotional and unspecified violence	During pregnancy; postpartum	BFI and EBF	BFI = 1.10 EBF = 1.41	No for BFI, but Yes for EBF P-value NR	Overall, P-IPV was not significantly associated with delayed initiation of breastfeeding but was significantly linked to a shorter duration of EBF.
Viana et al. (2024) [70]	Brazil	413	Cross-sectional	Physical, sexual, and Psychological /Emotional	During pregnancy	BFI	1.09	No, 0.868	The study did not find a significant association between P-IPV and delayed breastfeeding initiation.
Wallenborn et al. (2018) [47]	USA	195,264	Cross-sectional	Physical	Pre-pregnancy; during pregnancy	BFD	1.02	No, NR	No significant association was found between P-IPV and breastfeeding duration.

*BFI *breastfeeding initiation, *EBF* exclusive breastfeeding, *BFD* breastfeeding duration, *NR* Not reported

The studies used different tools to measure P-IPV (Table S4). The two most commonly used instruments were the Conflict Tactics Scale [40, 45, 50, 72, 74, 75] and the WHO violence against women instrument, each used in 6 out of 19 studies (31%) [6, 44, 70, 76–78]. Other tools included the composite abuse scale [43], the abuse assessment screen [21], the index of spouse abuse [42], and the domestic violence module from the Demographic and Health Survey (DHS) [71]. Additionally, one study [73] used the Pregnancy Risk Assessment Monitoring System (PRAMS) core questionnaire, while another [69] assessed P-IPV with a single-question measure: *“Someone hit*,* slapped*,* kicked*,* or injured you during pregnancy.”* One study did not specify the tool used to measure IPV during the perinatal period [47].

### Timing of IPV

IPV was assessed at different time points within the perinatal period. Five studies investigated IPV during the preconception (one year before pregnancy) and antenatal periods [21, 42, 47, 73, 77], while four measured IPV during both the antenatal and postpartum periods [6, 45, 72, 74], and two studies assessed IPV from one year pre-conception through to one year postpartum [44, 75]. The remaining studies focused exclusively on either during pregnancy [40, 50, 69, 70, 76, 78] or the postpartum period [43, 71].

### Breastfeeding outcomes ascertainment

The breastfeeding outcomes were assessed predominantly (*n* = 17) using self-reported data, with only two studies using hospital records to define them (**Table ****S5**). The types of breastfeeding outcomes reported were considerably varied across the studies. Of the included studies, only one assessed all three breastfeeding outcomes [45].

### Early initiation of breastfeeding

Ten studies [6, 21, 40, 42, 43, 45, 69, 70, 72, 73] reported outcomes based on the initiation of breastfeeding within one hour of birth. Among these, three studies [21, 69, 73] specifically focused on physical P-IPV, while seven studies [6, 40, 42, 43, 45, 70, 72] assessed all three common types of P-IPV (sexual, physical, and psychological).

### Exclusive breastfeeding

Thirteen studies [6, 21, 44, 45, 50, 69, 71, 72, 74–78] explored the relationship between P-IPV and EBF. Of these, four studies [21, 50, 69, 75] specifically examined physical P-IPV, while seven [44, 45, 71, 72, 74, 77, 78] assessed all three types of P-IPV (physical, sexual, and psychological). Two studies [6, 76] considered all three types of P-IPV alongside unspecified violence.

### Duration of breastfeeding

Four studies [43, 45, 47, 73] investigated the duration or continuation of breastfeeding in the context of P-IPV exposure. Among these, two studies [47, 73] specifically evaluated physical P-IPV, while two [43, 45] assessed all three types of P-IPV (physical, sexual, and psychological).

### Methodological risk of bias assessment

A methodological risk-of-bias assessment was conducted for all 19 studies included in this review (Table S6). Of these, 14 were classified as having a low risk of bias [6, 21, 40, 43, 44, 47, 50, 69–72, 74, 77, 78]. The remaining four studies had a moderate risk of bias [42, 45, 73, 76], and one was judged to have a high risk of bias [75]. This study did not clearly report the prevalence of early EBF interruption by exposure status or specify the reference groups for the reported effect estimate. Three cohort studies [42, 75, 76] did not clearly report whether the comparison groups were similar and recruited from the same population, resulting in an ‘Unclear’ rating for the first item of the critical appraisal checklist. Only two studies [69, 77] identified a minimally sufficient set of confounders using the DAG, while the remaining studies adjusted for various confounders without addressing all key factors. One study neither reported an adjusted effect estimate nor accounted for any confounding factors [70]. Overall, there was substantial variation in confounder adjustment across the included studies, ranging from two [76] to 17 variables [42]. Maternal age was the most commonly adjusted for variable (*n* = 15) [6, 21, 40, 42–44, 50, 69, 71–75, 77, 78], followed by maternal educational status (*n* = 14) [6, 42–44, 47, 50, 69, 71–75, 77, 78] (Table S5).

### Narrative review

Two studies [75, 76] that were not suitable for meta-analysis were included in the narrative analysis. A cohort study involving 564 mother-infant pairs who attended four primary health clinics in Rio de Janeiro, Brazil, found no statistically significant association between exposure to P-IPV and early interruption of EBF (ARR: 1.23, 95% CI: 0.99, 1.52) [75]. In contrast, another prospective cohort study involving 148 women in Vietnam found women exposed to P-IPV had a 30% higher absolute risk of early cessation of EBF compared to women not exposed to P-IPV (ARD: 0.3, 95% CI: 0.1, 0.5) [76]. As the study reported only an absolute risk difference from an Augmented Inverse Probability Weighting (AIPW) analysis, and insufficient information was available to derive a relative risk estimate, it was included in the narrative synthesis rather than the meta-analysis.

### Meta-analysis of the association between P-IPV and breastfeeding outcomes

A total of 17 studies were included in the meta-analysis. The pooled association between overall P-IPV and breastfeeding outcomes was examined, as were the associations between each type of P-IPV (physical, psychological, and sexual) and breastfeeding initiation, EBF, and breastfeeding duration.

### Association between P-IPV and EBF

**Fig. 2:**
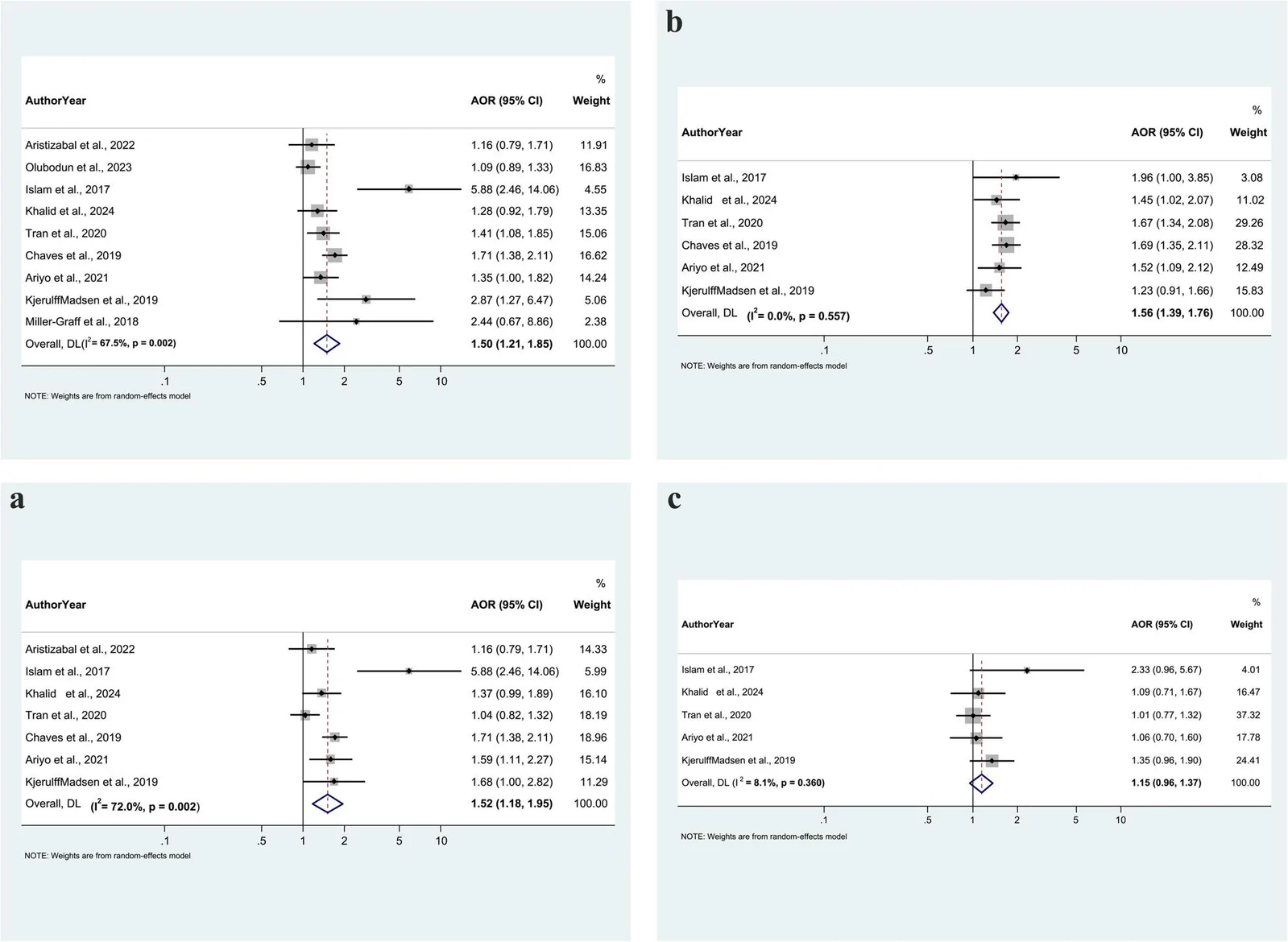
Forest plot of the association between P-IPV and EBF. **a**: Forest plot of the association between physical P-IPV and EBF. **b**: Forest plot of the association between psychological P-IPV and EBF. **c**: Forest plot of the association between sexual P-IPV and EBF

Nine studies with 40, 040 participants were analysed (Fig. 2). Five studies [6, 21, 71, 74, 78] identified a significant association between P-IPV and EBF, while the remaining four [44, 45, 69, 72] found no such association. The pooled estimate indicated a significant association, with women who experienced P-IPV having 1.50 times higher odds of not EBF compared to women not exposed (AOR: 1.50, 95%CI: 1.21, 1.85). In the subgroup analysis by study design, cohort studies reported a stronger association between P-IPV and EBF, with a pooled OR of 1.78 (95% CI: 1.46–2.18) and no heterogeneity (I² = 0.0%, *p* = 0.43). Cross-sectional studies showed a weaker but still significant association (OR = 1.36, 95% CI: 1.08–1.72), with substantial heterogeneity (I² = 66.7%, *p* = 0.01) (Table S7). Egger’s test indicated statistically significant evidence of small-study effects, but the trim-and-fill method suggested no missing studies, and the pooled effect size remained unchanged after adjustment, indicating no impact of publication bias (Figure S8). Two additional studies [60, 68] that reported AHRs instead of AORs found no significant association between P-IPV and EBF.

We also conducted subgroup meta-analyses to further explore the associations between specific types of P-IPV and breastfeeding outcomes. Seven studies reported associations with physical P-IPV, six with psychological P-IPV, and five with sexual P-IPV. The pooled result indicated a statistically significant association, with women exposed to physical P-IPV having 1.52 times higher odds of not EBF at six months compared to those not exposed (AOR: 1.52, 95% CI: 1.18, 1.95) (Fig. 2a). Similarly, we found a 56% increased risk of not exclusively breastfeeding among women exposed to psychological P-IPV (AOR: 1.56, 95% CI: 1.39–1.76) (Fig. 2b). However, no significant association was observed for sexual P-IPV (AOR: 1.15, 95% CI: 0.96–1.37) (Fig. 2c).

### Association between P-IPV and breastfeeding initiation

**Fig. 3:**
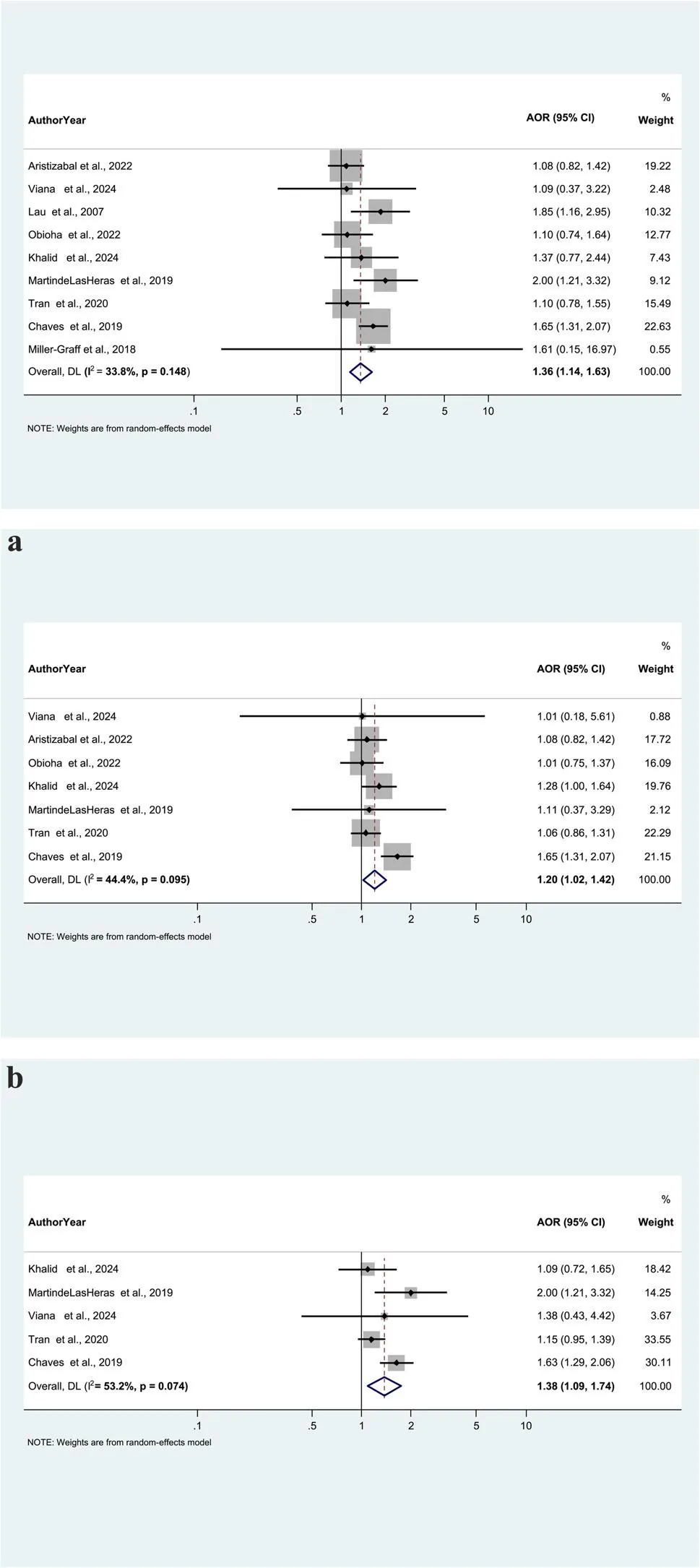
Forest plot of the association between P-IPV and breastfeeding initiation. **a**: Forest plot of the association between physical P-IPV and breastfeeding initiation. **b**: Forest plot of the association between psychological P-IPV and breastfeeding initiation

Nine studies involving 139,894 participants were included (Fig. 3). Three studies [21, 40, 42] reported a significant negative association between P-IPV and breastfeeding initiation, while six studies [6, 45, 69, 70, 72, 73] found no significant association. The pooled analysis showed that women exposed to P-IPV had 1.36 times higher odds of not initiating breastfeeding within one hour after childbirth compared to women not exposed to P-IPV (AOR: 1.36, 95% CI: 1.14, 1.63). With regard to specific types of P-IPV, both physical and psychological P-IPV were associated with delayed breastfeeding initiation. Physical P-IPV was associated with a 20% increase in the odds of delayed initiation (AOR: 1.20, 95% CI: 1.02–1.42) (Fig. 3a), and psychological P-IPV with a 38% increase (AOR: 1.38, 95% CI: 1.09–1.74) (Fig. 3b). However, we did not conduct a meta-analysis for sexual P-IPV due to an insufficient number of studies available.

### Association between P-IPV and breastfeeding duration

**Fig. 4:**
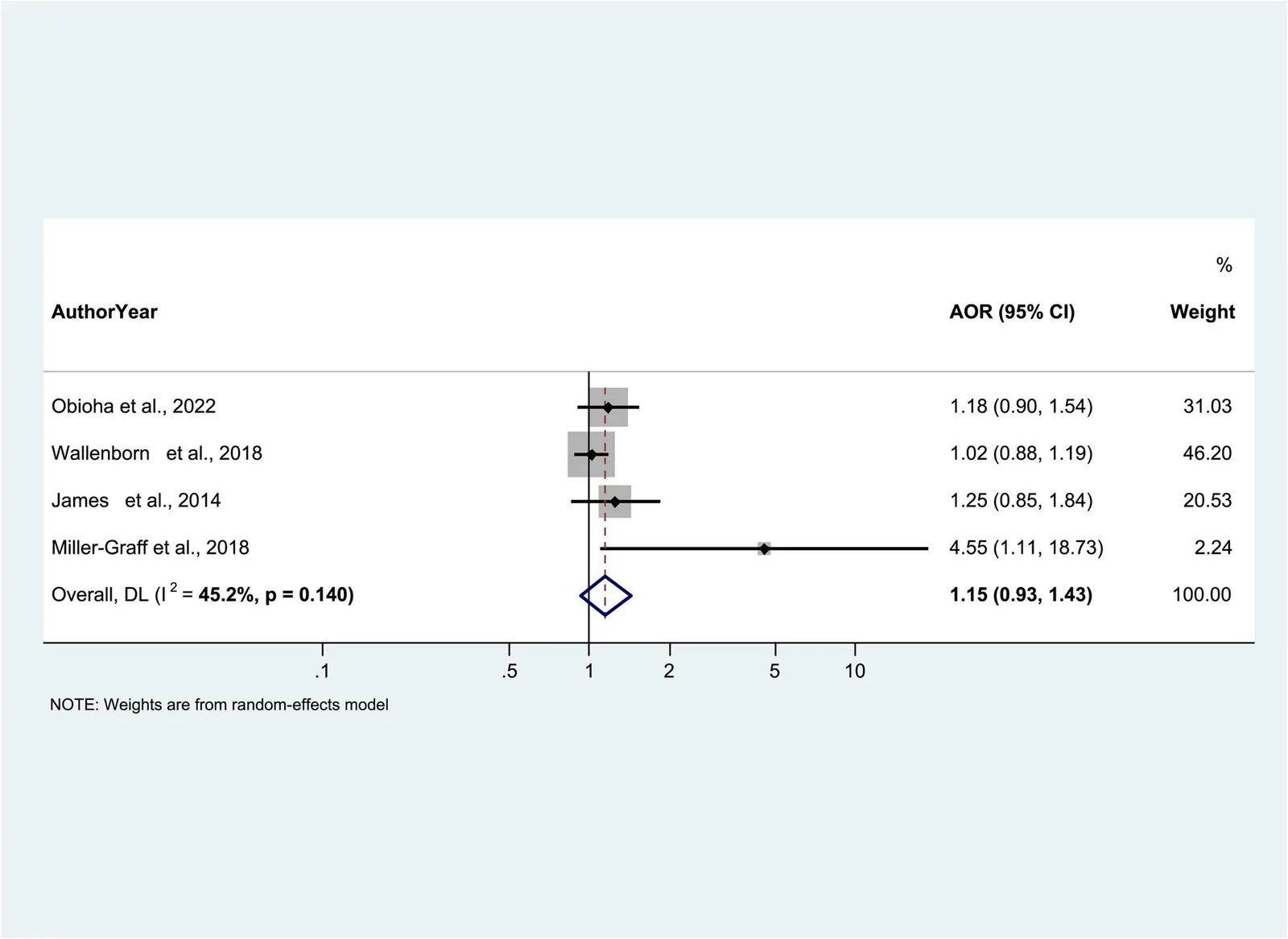
Forest plot of the association between P-IPV and breastfeeding duration

Four studies comprising 302,706 participants were included in the analysis (Fig. 4). Only one study [45] reported a significant association, while three [43, 47, 73] didn’t. The overall pooled estimate indicated no statistically significant association between P-IPV and breastfeeding duration (AOR: 1.15, 95% CI: 0.93, 1.43). Regarding specific types of P-IPV, only two studies [47, 73] examined the association between physical P-IPV and breastfeeding duration, and both reported no statistically significant association. No studies were identified that assessed the association between sexual or psychological P-IPV and breastfeeding duration. Due to the limited number of studies available, subgroup meta-analyses were not conducted for these exposures.

## Discussion

A P-IPV has been increasingly suggested as a potential barrier to optimal breastfeeding practices, potentially compromising infant and young child nutrition and development [79]. However, existing studies have reported inconsistent and conflicting findings regarding the relationship between P-IPV and breastfeeding outcomes. In response to these inconsistencies, our systematic review and meta-analysis synthesised the global evidence and provided a more comprehensive understanding of the association between P-IPV and breastfeeding outcomes. To the best of our knowledge, this is the first systematic review to quantitatively estimate the pooled effects of P-IPV on breastfeeding outcomes.

Building on this objective, we assessed the pooled association between overall and specific types of P-IPV and key breastfeeding outcomes, including initiation, exclusivity, and duration. Additionally, we examined the association between specific types of P-IPV (physical, psychological/emotional, and sexual) and each of these breastfeeding outcomes. Accordingly, the findings of this review indicate an association between P-IPV and poorer breastfeeding outcomes, with variations observed across different types of IPV and breastfeeding outcomes. Women who experienced IPV during the perinatal period, specifically, exposure to physical and psychological P-IPV, were more likely to delay breastfeeding initiation beyond one hour after childbirth and to discontinue EBF before six months, compared to those who did not experience IPV. However, no statistically significant association was observed between P-IPV and breastfeeding duration. We also found no evidence of an association between sexual P-IPV and EBF, although this finding was based on a limited number of studies.

Women exposed to P-IPV, specifically physical and psychological P-IPV, had 20% to 38% higher odds of delayed breastfeeding initiation compared to those who were not exposed. Several mechanisms may help explain the observed association between P-IPV and the delayed initiation of breastfeeding. P-IPV has been associated with increased maternal stress, anxiety, and depressive symptoms [80, 81], which may negatively affect breastfeeding initiation through both psychological and physiological pathways. Psychological distress during the perinatal period has been linked to poorer breastfeeding outcomes, including difficulties with initiating breastfeeding [82, 83]. From a physiological perspective, stress and anxiety can disrupt the neuroendocrine regulation of lactation by inhibiting oxytocin release, a key hormone in the milk ejection reflex, thereby making breastfeeding initiation more difficult in the immediate postpartum period [84]. Psychologically and behaviourally, P-IPV may impair maternal–infant bonding, reducing emotional connection with the newborn, which may, in turn, delay or hinder the initiation of breastfeeding within the first hour after birth [85]. It may also contribute to low self-efficacy, negative self-perception, self-doubt, and reduced emotional readiness, all of which can undermine a mother’s confidence to initiate breastfeeding within the first hour after birth [86, 87]. Physically, exposure to violence may result in pain, fatigue, or injury, which can limit a mother’s ability to initiate breastfeeding safely and comfortably immediately postpartum [54, 88]. In addition, P-IPV often occurs in the context of inadequate partner support, where the lack of emotional and practical assistance may reduce encouragement for timely breastfeeding initiation [89–93]. Finally, coercive behaviours that undermine women’s reproductive autonomy, including interference with infant feeding decisions, may further delay breastfeeding initiation [93, 94].

Moving beyond breastfeeding initiation, this review also revealed a significant negative association between P-IPV and EBF. Women exposed to P-IPV were less likely to exclusively breastfeed during the first six months; in particular, physical and psychological IPV increased the odds of non-exclusive breastfeeding by 52% and 56%, respectively. The observed association may be explained by various potential mechanisms. Firstly, mothers exposed to IPV, especially psychological abuse, often report lower breastfeeding self-efficacy, which diminishes their confidence in maintaining EBF [95]. Secondly, depression and anxiety, which are prevalent among IPV survivors [80, 96], can hinder maternal-infant bonding and reduce motivation to EBF [52, 82]. Lastly, the fear of further violence may cause mothers to prioritise their own physical and psychological safety and that of their infant, leading them to introduce formula or solid foods earlier than recommended by the WHO [97]. In contrast to these findings, no significant association between sexual P-IPV and EBF was observed. This may be partly explained by underreporting of sexual P-IPV, as women may be reluctant to disclose experiences of sexual violence due to stigma, shame, fear of negative consequences, and cultural taboos surrounding sexual abuse, which may have influenced the observed estimates of association [98, 99]. In addition, women exposed to sexual P-IPV often experience concurrent physical and psychological violence, making it difficult to isolate the independent effect of sexual P-IPV on EBF [100, 101].

The variation in the link between P-IPV and breastfeeding outcomes across different studies may partly reflect differences in sociocultural and healthcare settings. Norms around breastfeeding, gender roles, women’s autonomy, attitudes toward IPV, stigma, fear of disclosure, screening for IPV as a routine component of maternity care and access to maternity and child health services differ widely across countries and regions. These factors can influence both the likelihood of IPV disclosure and breastfeeding habits, contributing to differences in the observed associations [31, 102]. Recent research from 57 low- and middle-income countries shows significant variation in IPV prevalence and its determinants during pregnancy, emphasising the importance of contextual factors when interpreting results from diverse populations [11]. Additionally, qualitative evidence indicates that women experiencing P-IPV often face barriers to seeking help and may receive varying levels of support from health and social care services, which can impact maternal health behaviours, including breastfeeding practices [19]. Thus, understanding the relationship between P-IPV and breastfeeding outcomes requires considering the broader sociocultural and healthcare environment in which women live.

Turning to breastfeeding duration, our findings showed no significant association between P-IPV and breastfeeding duration, although this result is based on only four studies, mostly cross-sectional, that did not adjust for key confounders and had methodological limitations, including small sample sizes, inadequate follow-up, and poor handling of missing data.

Notably, there were insufficient studies examining the association between sexual P-IPV and breastfeeding initiation, as well as between psychological or sexual P-IPV and breastfeeding duration, precluding further meta-analysis for these exposures.

### Policy and practice implications

The findings of this review have important implications for clinical practice, maternal and child health services, and public health policy. Given the observed association between P-IPV and suboptimal breastfeeding outcomes, routine IPV assessment should be considered an integral part of perinatal care. Integrating trauma-informed and trauma-sensitive approaches into antenatal, intrapartum, and postnatal services may help healthcare providers identify women experiencing IPV and respond in ways that promote safety, trust, and supportive care. Healthcare professionals involved in maternity, newborn, and child care should be appropriately trained to recognise signs of IPV and understand its potential impact on breastfeeding. Women identified as experiencing P-IPV may benefit from tailored breastfeeding support that addresses both practical breastfeeding challenges and the psychological consequences of exposure to violence. The findings also highlight the importance of establishing referral pathways among maternity services, breastfeeding support programs, mental health services, social services, and IPV support organisations. Such integrated models of care may facilitate timely access to support and help address barriers that women experiencing P-IPV may face when initiating and maintaining recommended breastfeeding practices. At the policy level, strategies to improve breastfeeding outcomes should recognise P-IPV as an important social determinant of maternal and child health. Strengthening routine IPV screening, enhancing provider training, and ensuring access to coordinated support services may improve maternal well-being and infant feeding outcomes.

### Strengths and limitations

This review has certain strengths. It examined both overall and individual types of P-IPV (physical, psychological, and sexual) in relation to distinct breastfeeding outcomes (breastfeeding initiation, EBF, and BF duration). Importantly, the review covered the entire perinatal period (12 months before conception, throughout pregnancy, and up to 12 months postpartum), enabling a comprehensive assessment of IPV exposure. Furthermore, standardised definitions of breastfeeding outcomes, aligned with WHO indicators, were used to minimise heterogeneity across studies and enhance comparability.

Despite the aforementioned strengths, this review has certain limitations that should be considered when interpreting the results. Firstly, restricting the search to English-language articles may have omitted relevant studies published in other languages. Secondly, both P-IPV exposure and breastfeeding practices were commonly measured through self-report, which may have introduced recall bias. In particular, P-IPV may be underreported due to women’s unwillingness or inability to disclose their experiences, shaped by factors such as shame, embarrassment, guilt, fear of retaliation, a desire to maintain family unity, and financial dependency. Thirdly, the tools used to measure P-IPV varied across the included studies. This variation may have affected the comparability of exposure assessment and contributed to heterogeneity in the pooled estimates. Fourthly, methods used to ascertain breastfeeding outcomes also varied across studies. While most studies relied on self-reported data, others used hospital records. This variation may have affected the comparability of outcome assessment and contributed to heterogeneity in the findings. Fifthly, although the majority of studies were rated as high quality, many did not account for key confounding variables, which may have influenced the findings. Sixthly, the majority of articles included in this review were cross-sectional, which may limit the ability to assess the temporal relationship between P-IPV and breastfeeding outcomes. This is supported by our subgroup analyses (Tables S7 and S9), in which cohort studies showed a stronger and more consistent association than cross-sectional studies. Seventhly, the interpretation of the findings on sexual P-IPV is limited by the small number of available studies, small sample sizes, predominantly cross-sectional designs, and low methodological quality across the included studies. Lastly, this review was limited to studies involving heterosexual couples, which may limit the applicability of the findings to other relationship types, including same-sex and non-binary partnerships.

## Conclusions

In conclusion, this review provides evidence that overall P-IPV, and specifically physical and psychological P-IPV, are associated with delayed breastfeeding initiation and a lower likelihood of EBF at six months postpartum. We found no evidence of an association between P-IPV and breastfeeding duration. Similarly, the evidence regarding the association between sexual P-IPV and EBF was inconclusive. The findings highlight the potential value of trauma-informed and trauma-sensitive screening, support, and referral pathways for women experiencing P-IPV. Further high-quality studies are needed to investigate the association between P-IPV and breastfeeding duration, as well as the association between sexual P-IPV and breastfeeding outcomes.

## Supplementary Information

Supplementary Material 1.

Supplementary Material 2.

Supplementary Material 3.

Supplementary Material 4.

Supplementary Material 5.

Supplementary Material 6.

Supplementary Material 7.

Supplementary Material 8.

Supplementary Material 9.

## Data Availability

No datasets were generated or analysed during the current study.

## Abbreviations

AHR: Adjusted Hazard Ratio
AOR: Adjusted Odds Ratio
ANC: Antenatal Care
CI: Confidence Interval
COR: Crude Odds Ratio
CTS: Conflict Tactics Scale
DAG: Directed Acyclic Graph
DHS: Demographic and Health Survey
EBF: Exclusive Breastfeeding
IPV: Intimate Partner Violence
IYCF: Infant and Young Child Feeding
JBI: Joanna Briggs Institute
LBW: Low Birth weight
OR: Odds Ratio
PICO: Population, Intervention, Control, Outcomes
P-IPV: Perinatal Intimate Partner Violence
PRAMS: Pregnancy Risk Assessment Monitoring System
PRISMA: Preferred Reporting Items for Systematic Reviews and Meta-Analyses
PTSD: Post-Traumatic Stress Disorder
SD: Standard Deviation
SDG: Standard Deviation
SGA: Small for Gestational Age
STIs: Sexually Transmitted Infections
STROBE: Strengthening the Reporting of Observational Studies in Epidemiology
TNF-α: Tumor Necrosis Factor alpha: WHO: World Health Organisation
