# Supplementary material for: Association between perinatal intimate partner violence and breastfeeding outcomes: a systematic review and meta-analysis
Source: BMC Pregnancy Childbirth. 2026 Jul 27;26:1203. doi: 10.1186/s12884-026-09737-3 (PMC13688786; doi:10.1186/s12884-026-09737-3)
Supplement: Supplementary file 3 [file 12884_2026_9737_MOESM4_ESM.docx]

**Table S4:** Overview of included studies: Prevalence of P-IPV and Participant Characteristics

| **Author (s) (publication year)** | **Prevalence of P-IPV (%)** | **Tool used to measure P-IPV** | **Setting** | **Age** | **Educational status** | **Socioeconomic status** | **Marital status** |
| --- | --- | --- | --- | --- | --- | --- | --- |
| Aristizabal et al. (2022) | 6.40 | Single question: Someone hit, slapped, kicked or injured you during pregnancy | Household | Mean age=27.9 Yrs | Women's schooling: Primary =25.5%; Secondary =53.5%; Higher =19.2% | Wealth index: Poorest =29.4; Poorer =27.5; Middle =20.7  Richer =13.8; Richest= 8.6 | Never married =12.3%;  Currently married=71.9;  Formerly married =15.8 |
| Ariyo et al. (2021) | All three =21.1  Psychological = 16.6  Sexual= 4.6  Physical =11.7 | CTS | Households | Mean age =28 Yrs | The average years of education attained was 5 years | The mean household wealth index is -0.20 (SD =1.0) | All married women |
| Chaves et al. (2019) | 1.8 | AAS | Hospital | <20 Yrs cases=787 and participants= 27, 128  20-39 Yrs cases=60  and participants= 1497  >40 Yrs cases =12 and participants =448 | NR | Wealth index  High =394 (cases) and participants= 11,411  Middle= 380 (cases) and (participants)=14,370  Low= 85 (cases) and (participants) = 3292 | NR |
| Do et al. (2022) | 14.19 | WHO violence against women instrument | ANC clinic | 30 Yrs = 47.3% | Lower than high school = 33.8% | NR | NR |
| Hasselmann et al. (2016) | 33.7 | CTS | Primary health clinics | ≥ 20 Yrs =74.6%  <20 Yrs =25.4% | <8 years =52.0  ≥8 years= 48.0 | ≤10 household asset= 34.6%  >10 household asset= 65.4% | NR |
| Islam et al. (2017) | Physical after childbirth= 32.2  Sexual after childbirth=15.2  Psychological after childbirth= 60.8 | Domestic violence module from the demographic and health survey | Vaccination clinic | Age range of  15 –49 Yrs | No formal education =8.2%  Primary =24.4  Secondary and higher = 67.4% | ≤8500 =38.3%  >8500= 61.7% | 100% married (eligibility  for participation) |
| James et al. (2014) | 6.3 | Composite abuse scale. | Community health clinic and home | 15-24 Yrs =2.3%  25-29 Yrs =14.7%  30-34 Yrs =39.8%  35+ Yrs = 43.1% | Up to secondary = 13.1%  Diploma/apprenticeship = 23%  Degree/higher degree = 63.9% | Income  <$30,000=8.4%  $30,001-50,000=9.9%  $50,001-70,000=17.7%  >$70,000=64% | Married = 78.91%  De facto = 18.04%  Divorced/separated = 0.91%  Single/not living with  partner = 2.14% |
| Khalid et al. (2024) | Physical =47.44  Sexual =30.23  Emotional/psychological =51.72 | CTS | Household | Mean age of 27.7 Yrs | Average years of education =4.5 years | Poorest =20.74%  Poorer =23.09%  Middle =20.49%  Richer =16.71%  Richest= 18.98% | All married women |
| KjerulffMadsen et al. (2019) | Emotional = 23.5  Physical = 8.2  Sexual = 17.5  At least one kind = 29.5  All three = 4.0% | WHO violence against women instrument | ANC clinic | <20 Yrs = 12.1%  20-30 Yrs = 65.6%  >30 Yrs = 22.3% | Below primary education = 2.1%  Primary = 64.3%  Secondary = 28.6%  Above secondary = 5.0% | NR | Married = 89.6%  In a relationship  but not married = 10.3% |
| Lau et al. (2007) | Any IPV= 11.2  Psychological= 8.8  Sexual = 5.5  Physical = 4.1 | CTS | Hospital | >25 Yrs =80.4%  ≤25 Yrs =19.6% | NR | NR | Married =94.5% |
| MartindeLasHeras et al. (2019) | Psychological =21 | Index of spouse abuse | Hospital | <20 Yrs = 3.7%  20 - 24 Yrs = 13.6%  25 - 29 Yrs = 26.8%  30 - 34 Yrs = 37.2%  35 - 39 Yrs = 14.9%  ≥40 Yrs = 3.7% | Years of schooling:  <7 years = 36.5%  7 - 12 years = 48.8%  >12 years = 14.6% | NR | Married = 65.1%  Committed = 14.2%  Non-committed = 20.7% |
| Miller-Graff et al. (2018) | 37.7 | CTS | WIC service - prenatal appointment | Mean age of 26.5 Yrs | NR | On average, monthly household income was $1,149.55 (SD= 927.22) | Most women were  single or  divorced (n = 39, 55.72%) |
| Moraes et al. (2011) | 18.9 | CTS | Community health centre | <20 Yrs = 22.7%  20 - 35 Yrs = 68.3%  >35 Yrs = 9.0% | Primary school incomplete = 41.4%  Primary school completed= 30.4%  Secondary school completed = 21.4%  Higher education, whether completed or not = 6.7% | Brazilian criterion of economic classification  A (highest) = 1.9%  B = 10.0%  C = 45.6%  D = 40.7%  E (lowest) = 1.9% | NR |
| Obioha et al. (2022) | Pre-pregnancy = 1.6  Prenatal =1.4 | PRAMS core questionnaire | Household | 17-19 Yrs = 4.1%  20-34 Yrs =77.6%  ≥35 Yrs =18.3% | ≤ High school (<13 years) =38,492 (36.3%)  > High school (13 years+) =65,684 (63.7%) | Lower class (≤28,000) = 37.9%  Working class ($28,001-$40,000) =10.7%.  Middle class ($40,001-$100,000) = 24.0%.  Upper class (≥$100,001) =27.4% | Married =62.7%  Other =37.3% |
| Olubodun et al. (2023) | Emotional = 28  Physical= 13.4  Sexual = 5  Any IPV = 31.1 | WHO violence against women instrument | Household | 15-19 Yrs = 8.5%  20-29 Yrs = 47.1%  30-39 Yrs = 39.0%  40-49 Yrs = 5.4% | No education = 40.2%  Primary = 13.7%  Secondary = 33.4%  Higher = 12.7% | Household wealth quintile  Poorest = 18.5%  Poorer = 20.7%  Middle = 18.3%  Richer = 21.1%  Richest = 1.4% | NR |
| RibeiroMRC et al. (2021) | Psychological =47.5  Physical =12.1  Sexual =2.8 | WHO violence against women instrument | Prenatal services and ultrasound clinics | Median age of  25 Yrs | 76% had 11 years of schooling | 68% belonged to socioeconomic class C | Lives with husband/  partner =79% |
| Tran et al. (2020) | All three =11.0  Any IPV =49.7  Sexual =19.3  Physical =28.7  Emotional =39.1 | WHO violence against women instrument | Household | Age range of 13-44 Yrs | No education=11.6%  Elementary school =35.15%  Middle school= 37.9%  High school or higher 15.35% | Household food insecurity=891(44.6%)  SES (tertile)= 655 (33.3%) | NR |
| Viana et al. (2024) | During pregnancy  Any IPV =3.6  Physical= 1.5  Psychological =3.6  Pre-pregnancy  Physical =93  Psychological =89.3  Sexual =99.3 | WHO violence against women instrument | Hospital | Mean age of 27.1 Yrs | Years of schooling:  0-4 years= 3.1%  5-8 years = 24.5%  9-11 years = 62.7%  ≥12 years = 9.7% | Have income =75 (18.2%)  Have no income= 338 (81.8%) | With partner =86.0%  Without partner =14.0% |
| Wallenborn et al. (2018) | Pre-pregnancy and/or during pregnancy=5.3  Pre-pregnancy and during pregnancy=2.6 | Not reported | Household | <20 Yrs = 12.3%  20-24 Yrs = 28.7%  25-29 Yrs = 27.8%  30-34 Yrs = 19.7%  35+ Yrs =11.5% | **<**12 years =20.3%  High school diploma =33.8%  >12 years =45.9% | Private =36.7%  Medicaid =44.7%  None =2.7%  Other =2.2%  Multiple =13.7% | Unmarried =49.6%  and the majority  were married |
| ANC, antenatal care; NR, not reported; Yrs, years, AAS = Abuse Assessment Screen; CTS = Conflict Tactics Scale; PRAMS = Pregnancy Risk Assessment Monitoring System;  WHO = World Health Organization | | | | | | | |
