# Supplementary material for: Association between perinatal intimate partner violence and breastfeeding outcomes: a systematic review and meta-analysis
Source: BMC Pregnancy Childbirth. 2026 Jul 27;26:1203. doi: 10.1186/s12884-026-09737-3 (PMC13688786; doi:10.1186/s12884-026-09737-3)
Supplement: Supplementary file 4 [file 12884_2026_9737_MOESM5_ESM.docx]

**Table S5:** Summary of confounders adjusted for and statistical models used in the included studies

| **Author (s) (year)** | **Definition of breastfeeding** | **Statistical model/analysis used** | **Confounder variables adjusted for** |
| --- | --- | --- | --- |
| Aristizabal et al. (2022) | Self-reported | Propensity Score Analysis using Inverse Probability of Treatment Weighting (IPTW), with propensity scores estimated through Generalised Boosted Modelling (GBM). Effect estimates were reported as Odds Ratios (ORs) with 95% Confidence Intervals (CIs). Sensitivity analysis was conducted using Rosenbaum bounds (Gamma statistic). | Maternal age, maternal education level, maternal marital status, women’s race, maternal occupation, wealth index, partner age, partner schooling, alcohol and drug consumption by partner, number of children, pregnancy planning, type of family |
| Ariyo et al. (2021) | Self-reported | Weighted multivariable logistic regression analysis (complete-case and multiple-imputation models using Markov Chain Monte Carlo [MCMC] imputation), reporting adjusted odds ratios (AORs) and 95% confidence intervals. | Maternal age, maternal education, partner education, employment status, wealth index, ANC visit, residence, perceived size (birth weight), parity, child age, no of under 5 children |
| Chaves et al. (2019) | Hospital records | Multivariable logistic regression; MICE multiple imputation (20 imputations); ORs with 95% CIs. | Maternal age, socio-economic status, indigenous status, alcohol consumption during pregnancy, smoking during pregnancy, body mass index, history of antenatal problems, antenatal depression, and intervention during birth. |
| Do et al. (2022) | Self-reported | Hierarchical multivariable logistic regression; AIPW (Augmented Inverse Probability Weighting) models; E-value sensitivity analysis; Structural Equation Modeling (SEM) with bootstrapped maximum likelihood estimation (1000 replications). | Childhood abuse and lifetime major depression |
| Hasselmann et al. (2016) | Self-reported | Multivariable Poisson regression with robust variance; adjusted prevalence ratios (PRs) and relative risks (RRs) with 95% CIs. | Infant’s sex, infant’s age (in days), birth weight, household assets, maternal schooling, and maternal age |
| Islam et al. (2017) | Self-reported | Multivariable logistic regression (AORs, 95% CIs); VIF-based multicollinearity assessment; interaction analysis using binary logistic regression. | Maternal education, residence, family structure, family monthly income, parity, maternal health status, age of child, gender of the last child, number of children under five, pregnancy intention, number of ANC visits, delivery mode, place of delivery, complication during child birth, postpartum depression, social support, maternal age at last pregnancy |
| James et al. (2014) | Self-reported | Binary multivariable logistic regression adjusted for socio-demographic and obstetric covariates; AORs with 95% CIs; cluster-adjusted standard errors; contingency table analyses. | Maternal age, maternal education, income, health care card, mode of delivery, parity and preterm birth. Also, adjustment for clustering by site. |
| Khalid et al. (2024) | Self-reported | Survey-weighted multivariable logistic regression; MCMC multiple imputation (11 imputations); AORs with 95% CIs; sensitivity analysis using logistic regression; VIF-based multicollinearity assessment. | Maternal age, maternal education, children age, maternal employment, number of children, husband’s education, residence, family wealth index, number of under-5 children |
| KjerulffMadsen et al. (2019) | Self-reported | Multivariable logistic regression (adjusted ORs, 95% CIs); DAG-guided confounder selection; stratified logistic regression analyses. | Maternal age, education, HIV status, alcohol use during pregnancy and parity |
| Lau et al. (2007) | Self-reported | Multinomial logistic regression with adjusted odds ratios (AORs) and 95% CI. | Maternal age, parity, place of birth, marital status, living condition, breastfeeding support, pregnancy intention, mode of delivery |
| MartindeLasHeras et al. (2019) | Hospital records | Multivariable logistic regression (aORs, 95% CIs). | Maternal age, civil status, maternal education, maternal occupation, maternal nationality, cohabitation, kin support, desired pregnancy, obstetric pathologies, infection during pregnancy, infant sex, preterm birth, low birth weight, resuscitation and smoking during pregnancy |
| Miller-Graff et al. (2018) | Self-reported | Multivariable logistic regression (ORs, 95% CIs); bias-corrected logistic regression for unequal cell sizes. | Perinatal health problems, prenatal depression, childhood adverse experiences, and prenatal breastfeeding education |
| Moraes et al. (2011) | Self-reported | Complementary log–log regression (current-status survival analysis/Weibull equivalent) with hierarchical multivariable modelling; HRs and 95% CIs. | Mothers educational level, mothers working during postpartum period, wealth index based on brazilian criterion of economic classification, child age (in days), mothers age, desired pregnancy, alcohol consumption by mother, mother smoking, mother illicit drug use, gestational age at birth |
| Obioha et al. (2022) | Self-reported | Multivariable logistic regression (ORs, 95% CIs); interaction analysis using likelihood ratio tests; complex survey-adjusted analysis. | Maternal age, marital status, annual household income, maternal education level, residence, race, ethnicity |
| Olubodun et al. (2023) | Self-reported | Survey-weighted multivariable logistic regression (aORs, 95% CIs). | Woman’s age, woman’s educational level, employment status, household wealth quintile, number of antenatal care visits, parity, child’s age group, sex of the child, place of childbirth, mode of childbirth, type of place of residence, and region |
| RibeiroMRC et al. (2021) | Self-reported | Kaplan–Meier survival analysis, log-rank test, and multivariable Cox proportional hazards regression (HRs, 95% CIs); DAG-based confounder selection. | Person with highest income in family, economic class, pregnant woman's age, woman's education, partner's age, lives with partner, alcohol in pregnancy, social support, skin colour, number of male intimate partners, religion, paid job, number of children, planned pregnancy, breastfeeding decision |
| Tran et al. (2020) | Self-reported | Multivariable logistic regression (ORs, 95% CIs) and path analysis/Structural Equation Modelling (SEM) to assess direct and indirect pathways. | Maternal age, maternal education, early marriage, parity, autonomy, socioeconomic status, food security, spousal education gap, child age, child gender, geographical clustering, maternal common disorder. |
| Viana et al. (2024) | Self-reported | Hierarchical multivariable logistic regression (AORs, 95% CIs); Pearson's Chi-square/Fisher's exact tests for bivariate analyses. | Not reported (no confounding adjusted for) |
| Wallenborn et al. (2018) | Self-reported | Multinomial logistic regression (AORs, 95% CIs). | Marital status, maternal education, and insurance status |
