# Supplementary material for: Association between perinatal intimate partner violence and breastfeeding outcomes: a systematic review and meta-analysis
Source: BMC Pregnancy Childbirth. 2026 Jul 27;26:1203. doi: 10.1186/s12884-026-09737-3 (PMC13688786; doi:10.1186/s12884-026-09737-3)
Supplement: Supplementary file 5 [file 12884_2026_9737_MOESM6_ESM.docx]

**Table S6:** Summary of the design-specific critical appraisal results

| **Study design** | **Critical appraisal questions and their response** | | | | | | | | | | | **The overall risk of bias** |  |
| --- | --- | --- | --- | --- | --- | --- | --- | --- | --- | --- | --- | --- | --- |
| **Cohort study** |  |  |  |  |  |  |  |  |  |  |  |  |  |
| **Author and year** | **Q1** | **Q2** | **Q3** | **Q4** | **Q5** | **Q6** | **Q7** | **Q8** | **Q9** | **Q10** | **Q11** |  |  |
| Chaves et al. (2019) | Y | Y | Y | N | Y | Y | Y | Y | U | Y | Y | Low | |
| Do et al. (2022) | U | U | Y | N | Y | Y | Y | Y | Y | NA | Y | Moderate | |
| Hasselmann et al. (2016) | U | Y | Y | N | Y | U | U | Y | N | N | Y | High | |
| James et al. (2014) | Y | Y | Y | N | Y | Y | Y | Y | U | U | Y | Low | |
| KjerulffMadsen et al. (2019) | Y | U | Y | N | Y | Y | Y | Y | Y | U | Y | Low | |
| MartindeLasHeras et al. (2019) | U | U | Y | N | Y | Y | Y | Y | U | U | Y | Moderate | |
| Miller-Graff et al. (2018) | Y | Y | Y | N | Y | Y | Y | N | N | N | Y | Moderate | |
| RibeiroMRC et al. (2021) | Y | Y | Y | Y | Y | Y | Y | N | Y | Y | Y | Low | |
| % | 63 | 63 | 100 | 13 | 100 | 88 | 88 | 75 | 38 | 25 | 100 |  | |
| **Analytical cross-sectional study** | **Q1** | **Q2** | **Q3** | **Q4** | **Q5** | **Q6** | **Q7** | **Q8** | **The overall risk of bias** | | | | |
| Aristizabal et al. (2022) | Y | Y | N | Y | Y | Y | Y | Y | Low | | | | |
| Ariyo et al. (2021) | Y | Y | Y | Y | N | Y | Y | Y | Low | | | | |
| Islam et al. (2017) | Y | Y | Y | Y | N | Y | Y | Y | Low | | | | |
| Khalid et al. (2024) | Y | Y | Y | Y | N | Y | Y | Y | Low | | | | |
| Lau et al. (2007) | Y | Y | Y | Y | N | Y | Y | Y | Low | | | | |
| Moraes et al. (2011) | Y | Y | Y | Y | N | Y | Y | Y | Low | | | | |
| Obioha et al. (2022) | Y | Y | U | Y | N | N | U | Y | Moderate | | | | |
| Olubodun et al. (2023) | Y | Y | Y | Y | N | Y | Y | Y | Low | | | | |
| Tran et al. (2020) | Y | Y | Y | Y | N | Y | Y | Y | Low | | | | |
| Viana et al. (2024) | Y | Y | Y | Y | N | Y | Y | Y | Low | | | | |
| Wallenborn et al. (2018) | Y | Y | U | Y | N | Y | Y | Y | Low | | | | |
| % | 100 | 100 | 73 | 100 | 9 | 91 | 91 | 100 |  | | | | |

N=no; Y=yes; U=unclear; NA=Not applicable.
