# Supplementary material for: Association between perinatal intimate partner violence and breastfeeding outcomes: a systematic review and meta-analysis
Source: BMC Pregnancy Childbirth. 2026 Jul 27;26:1203. doi: 10.1186/s12884-026-09737-3 (PMC13688786; doi:10.1186/s12884-026-09737-3)
Supplement: Supplementary file 6 [file 12884_2026_9737_MOESM7_ESM.docx]

| Variables | Subgroup | No of studies | Population (N) | OR (95%CI) | I² (%) | Cochrane's Q test p-value |
| --- | --- | --- | --- | --- | --- | --- |
| Study design | Cohort | 3 | 18,794 | 1.78 (1.46-2.18) | 0.0 | 0.43 |
|  | Cross-sectional | 6 | 20,803 | 1.36 (1.08-1.72) | 66.7 | 0.01 |
| Tool to measure IPV | CTS | 3 | 3757 | 1.34 (1.08-1.67) | 0.0 | 0.64 |
|  | WHO violence against women instrument | 3 | 6,877 | 1.38 (0.97-1.96) | 69.3 | 0.04 |
