# Supplementary material for: Association between perinatal intimate partner violence and breastfeeding outcomes: a systematic review and meta-analysis
Source: BMC Pregnancy Childbirth. 2026 Jul 27;26:1203. doi: 10.1186/s12884-026-09737-3 (PMC13688786; doi:10.1186/s12884-026-09737-3)
Supplement: Supplementary file 7 [file 12884_2026_9737_MOESM8_ESM.docx]

Figure S8: Funnel plot for IPV and BF initiation

Egger’s test for small study effects (p=0.866)

Figure S8a: IPV and EBF funnel plot and Egger’s test

Egger’s test for small study effects (p=0.019)

Trim and Fill output for IPV and EBF

| **Trim-and-fill analysis** | **Result** |
| --- | --- |
| Number of observed studies | 9 |
| Number of imputed studies | 0 |
| Observed pooled effect (95% CI) | 2.089 (1.116–3.061) |
| Adjusted pooled effect (Observed + Imputed) (95% CI) | 2.089 (1.116–3.061) |

**Figure S8b: IPV and BF duration**

Egger’s test for small study effects (p < 0.001)

| **Trim-and-fill analysis** | **Result** |
| --- | --- |
| Number of observed studies | 4 |
| Number of imputed studies | 0 |
| Observed pooled effect (95% CI) | 1.878 (0.377–3.380) |
| Adjusted pooled effect (Observed + Imputed) (95% CI) | 1.878 (0.377–3.380) |
