# Supplementary material for: Association between perinatal intimate partner violence and breastfeeding outcomes: a systematic review and meta-analysis
Source: BMC Pregnancy Childbirth. 2026 Jul 27;26:1203. doi: 10.1186/s12884-026-09737-3 (PMC13688786; doi:10.1186/s12884-026-09737-3)
Supplement: Supplementary file 8 [file 12884_2026_9737_MOESM9_ESM.docx]

| Variables | Subgroup | No of studies | Population (N) | OR (95%CI) | I² (%) | Cochrane's Q test p-value |
| --- | --- | --- | --- | --- | --- | --- |
| Study design | Cohort | 2 | 18,692 | 1.71 (1.40-2.07) | 0.0 | 0.95 |
|  | Cross-sectional | 5 | 17498 | 1.47 (1.05-2.07) | 75.6 | 0.003 |
